# Tackling Waste Polystyrene with Sunlight

**DOI:** 10.1021/acscentsci.4c02187

**Published:** 2025-01-02

**Authors:** Hyun Suk Wang, Athina Anastasaki

**Affiliations:** Laboratory of Polymeric Materials, Department of Materials, ETH Zurich, Vladimir-Prelog-Weg 5 8093, Zurich, Switzerland

You would not want to touch the surface of a black car on a sunny summer day—it is scorching! This is because of a phenomenon called “photothermal conversion,” or a light-to-heat conversion of black pigments on the car surface. In this issue of *ACS Central Science*, Stache and co-workers exploit this very phenomenon to chemically recycle polystyrene (PS) to its monomer, styrene, using an inexpensive and ubiquitous material—carbon black.^1^

PS, widely recognized for its use in packaging and disposable products, presents a significant environmental challenge due to its low recycling rates and accumulation in landfills. Although mechanical recycling methods exist, they are limited by the inevitable degradation of material properties with each cycle. Chemical recycling via depolymerization,^2,3^ which reverts PS back into styrene monomers, offers the potential for infinite recycling but is typically constrained by high energy demands, requiring temperatures typically in excess of 400 °C.^4^ Furthermore, bulk heating of PS leads to an uncontrolled flux of reactive intermediates, leading to undesirable byproducts. Photochemical methods for PS conversion have also been limited, as they often produce non-monomeric products (e.g., benzoic acid) due to the thermodynamic challenges of styrene depropagation.^5^

**Figure 1:**
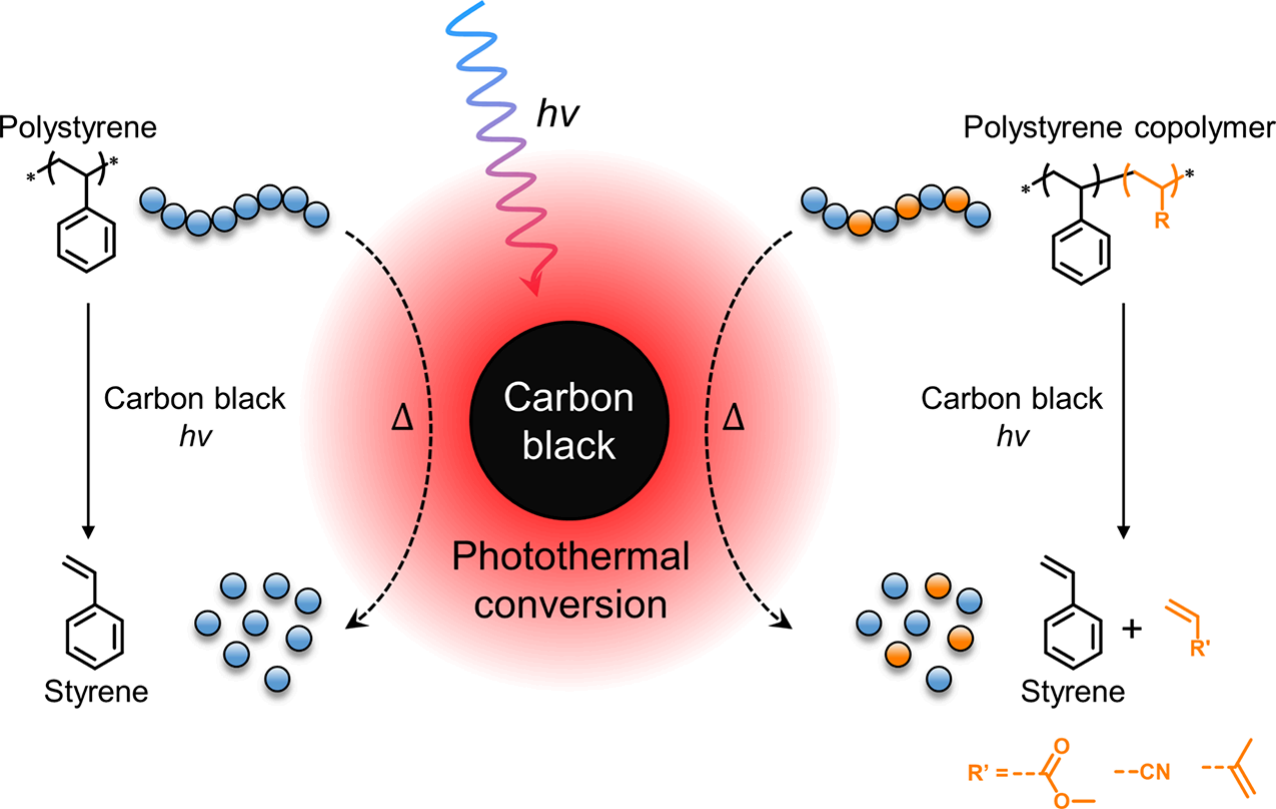
Depolymerization of polystyrene (co)polymers using carbon black as photothermal conversion agents, as developed by Stache and co-workers.^1^ Copyright 2024. The Authors. Published by American Chemical Society.

The authors present a remarkably simple approach that addresses these limitations by leveraging carbon black as a photothermal conversion agent. Their method achieves efficient depolymerization of PS under visible light irradiation, creating localized thermal hotspots while maintaining subpyrolytic bulk temperatures (Figure 1). Carbon black (CB) is a cost-effective pigment widely used in commercial products, such as coffee cup lids. However, its near-zero recycling rate makes it an environmental burden. Stache and colleagues have repurposed this ubiquitous material, utilizing its high photothermal conversion efficiency (i.e., low fluorescence quantum yield) that drives the depolymerization of PS. As a proof of concept, the authors synthesized PS particles embedded with varying amounts of CB via emulsion polymerization to maximize physical contact between the polymer and the photothermal agent. Upon irradiating these PS-CB composites with white LEDs, a maximum styrene yield of 57% was achieved after 30 min. Remarkably, the bulk temperature during the reaction remained below 150 °C, as measured with a thermocouple, despite the purely thermal nature of the process. This efficiency of depolymerization at low bulk temperatures is noteworthy, particularly when compared to conventional pyrolysis methods. Other products of the reaction, including trimers, dimers, toluene, and α-methylstyrene, accounted for approximately 30% of the total small-molecule products. Furthermore, CB could be reused for multiple depolymerization cycles, highlighting the robustness of the methodology.

The versatility of the CB-based depolymerization process was demonstrated through its application to various styrene-based copolymers containing comonomers with notoriously high ceiling temperatures (i.e., with low propensity for depropagation). Copolymers incorporating methyl acrylate, acrylonitrile, and isoprene were successfully depolymerized to regenerate both styrene and the comonomers, with only a minor reduction in overall conversion efficiency.

**Figure 2:**
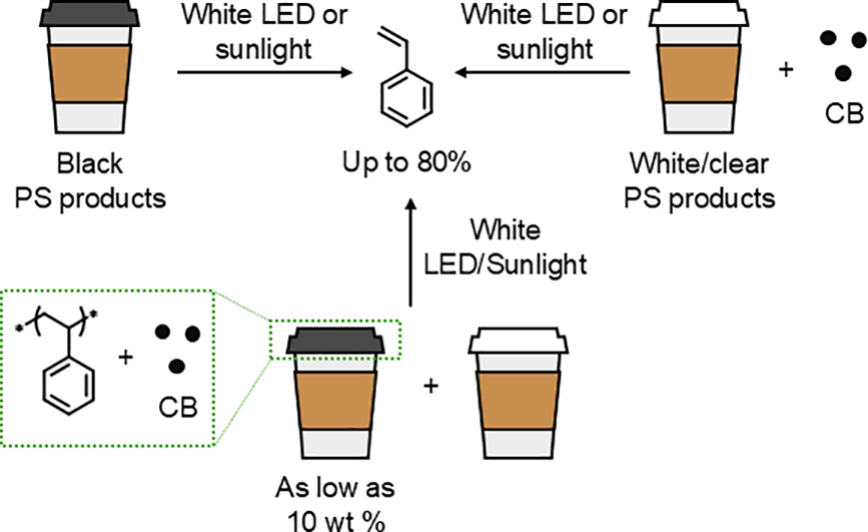
Depolymerization of commercial polystyrene products using either already-present black pigments or additional carbon black.

The methodology was further validated with postconsumer plastics. Black PS products, such as foam trays, food containers, and coffee cup lids—materials that already contain carbon black—were effectively depolymerized (Figure 2). Even white and clear PS plastics could be depolymerized after the addition of CB. Importantly, the reaction was robust against common food contaminants, such as soy sauce and sugar, maintaining high yields despite their presence. Additionally, mixed plastic waste streams containing as little as 10% by weight black PS foam achieved comparable yields. This suggested that black plastic contamination in waste streams could even be beneficial in enhancing recycling. In a demonstration of the process’s sustainability, the authors used sunlight instead of LEDs as the energy source. By focusing natural sunlight onto black PS foam using a Fresnel lens, they achieved an impressive styrene yield of 80%. Although the contribution of direct thermal effects from the concentrated sunlight is unclear, direct photolysis of the PS backbone by high-energy rays (λ < 300 nm) can be ruled out as the lens was made of plastic.

The work by the Stache team represents a significant advancement in chemical recycling, and the next step could potentially involve scaling the technology for industrial applications. Light penetration through carbon-black-filled plastics is inherently limited, necessitating the development of specialized reactor designs or extended reaction times to ensure consistent processing. Nevertheless, the simplicity and effectiveness of this method, coupled with its compatibility with current waste streams, position it as a promising candidate for future recycling technologies. By addressing technical challenges, this approach could pave the way for scalable, energy-efficient chemical recycling solutions.
